# Exosome-Containing Extracellular Vesicles Contribute to the Transport of Resveratrol Metabolites in the Bloodstream: A Human Pharmacokinetic Study

**DOI:** 10.3390/nu14173632

**Published:** 2022-09-02

**Authors:** Carlos Eduardo Iglesias-Aguirre, María Ángeles Ávila-Gálvez, María-Carmen López de las Hazas, Alberto Dávalos, Juan Carlos Espín

**Affiliations:** 1 Laboratory of Food & Health, Research Group on Quality, Safety, and Bioactivity of Plant Foods, Campus de Espinardo, CEBAS-CSIC, 30100 Murcia, Spain; 2 Instituto de Biologia Experimental e Tecnológica (iBET), Apartado 12, 2781-901 Oeiras, Portugal; 3 NOVA Medical School, Faculdade de Ciências Médicas (NMS|FCM), Universidade Nova de Lisboa, 1150-082 Lisboa, Portugal; 4 Laboratory of Epigenetics of Lipid Metabolism, Madrid Institute for Advanced Studies (IMDEA)-Food, CEI UAM + CSIC, 28049 Madrid, Spain

**Keywords:** exosome, extracellular vesicles, metabolites, pharmacokinetics, resveratrol, gut microbiota, lunularin, dihydroresveratrol, polyphenol

## Abstract

Exosomes are extracellular vesicles (EVs) that regulate intercellular signaling by transferring small RNAs, proteins, nucleic acids, lipids, and other metabolites to local or distant organs, including the brain, by crossing the blood–brain barrier. However, the transport of (poly)phenols in human EVs has not yet been described. Therefore, we aimed here to explore (i) whether resveratrol and (or) its derived metabolites are found in the cargo of human plasma exosome-containing EVs (E-EVs), (ii) when this incorporation occurs, and (iii) whether resveratrol intake stimulates the release of E-EVs. Thus, in a pharmacokinetic study, healthy volunteers (n = 16) consumed 1 capsule (420 mg resveratrol) in the evening before attending the clinic and one more capsule on the day of the pharmacokinetics. The plasma and the isolated E-EVs were analyzed using UPLC-ESI-QTOF-MS. Of 17 metabolites in the plasma, 9 were identified in the E-EVs, but not free resveratrol. The kinetic profiles of resveratrol metabolites were similar in the plasma and the E-EVs, a higher metabolite concentration being detected in the plasma than in the E-EVs. However, the plasma/E-EVs ratio decreased in the gut microbial metabolites, suggesting their better encapsulation efficiency in E-EVs. In addition, glucuronide conjugates of resveratrol, dihydroresveratrol, and lunularin were incorporated into the E-EVs more efficiently than their corresponding sulfates despite glucuronides reaching lower plasma concentrations. Notably, more E-EVs were detected 10 h after resveratrol consumption. This exploratory study provides the first evidence that (i) resveratrol metabolites are transported by E-EVs, with a preference for glucuronide vs. sulfates, (ii) the gut microbial metabolites concentration and kinetic profiles are closely similar in E-EVs and plasma, and (iii) resveratrol intake elicits E-EVs secretion. Overall, these results open new research avenues on the possible role of E-EVs in (poly)phenol health effects.

## 1. Introduction

Exosomes are a nanosized (30–150 nm) subclass of extracellular vesicles (EVs) containing specific membrane proteins (TSG101, CD63, and others). These intraluminal vesicles are formed by the inward budding of the endosomal membrane during the maturation of multivesicular bodies and are secreted from cells upon fusion of the multivesicular body (the endocytic compartment) with their surface membrane [1]. However, there is confusion regarding the precise term used to name these extracellular particles (EVs, exosomes, microvesicles, etc.), so EVs are also often referred to as exosomes upon detecting the corresponding membrane exosome biomarkers, according to the International Society of Extracellular Vesicles (ISEV) [2]. EVs are released by all cells and are widely distributed in biofluids. EVs participate as important regulators of intercellular signaling by transferring their cargo (small RNAs, proteins, metabolites, nucleic acids, and lipids) to target cells locally as a kind of paracrine signaling, or by traveling to a distant body site contributing to homeostasis and disease via diverse functions [3,4,5]. Thus, there is a growing interest in analyzing EVs from biofluids as a means of disease diagnosis and therapeutic monitoring [6,7].

(Poly)phenols have been acknowledged to have many biological properties exerted through multiple mechanisms [8,9,10]. For example, in the context of EVs, previous in vitro studies have shown that curcumin promotes exosome release to remove cholesterol accumulated within the endolysosomal compartment in cells with impaired intracellular cholesterol trafficking and induces ceramide synthesis de novo in glial cells [11,12,13]. In addition, resveratrol (RSV)-treated primary microglia cells release EVs capable of crossing the blood–brain barrier and restoring neural function by the induction of autophagy and inhibition of apoptosis of neurons via the PI3K signaling pathway [14]. However, the mechanism by which (poly)phenols modulate exosome secretion and cargo content is not fully understood [15].

EVs have emerged as nanocarriers of bioactive molecules, including (poly)phenols, to increase their bioavailability and bioactivity [16,17]. However, the possible presence of (poly)phenols in the plasma exosome cargo has been scarcely addressed. For instance, a rat study showed that exosome-containing EVs (E-EVs) minimally transported grape seed proanthocyanidins and derived metabolites [18]. While albumin, lipoproteins, and red blood cells can transport (poly)phenols in the human bloodstream [19,20,21,22], no studies have reported their possible transport in human EVs. In this regard, Vallejo et al. [7] failed to detect phenolic-derived metabolites using non-targeted metabolomics in the plasma exosome cargo of fasting healthy volunteers and patients at increased risk of thrombosis. Thus, it is unknown whether (poly)phenols and (or) derived metabolites are transported in human EVs in response to the dietary intake of (poly)phenols. Furthermore, if this were to occur, it would also be necessary to estimate when (poly)phenol incorporation into exosomes might occur.

Therefore, in the present pharmacokinetic study, we aim to test (i) whether RSV and (or) its derived metabolites are found in the cargo of human plasma EVs, (ii) the encapsulation kinetics in E-EVs, and (iii) whether RSV intake stimulates EVs release.

## 2. Materials and Methods

### 2.1. Chemicals and RSV Capsules

HPLC-grade acetonitrile, dimethyl sulfoxide (DMSO), formic acid, and methanol were obtained from JT Baker (Deventer, The Netherlands). *Trans*-Resveratrol (3,5,4′-trihydroxy-*trans*-stilbene, resveratrol, RSV, ≥99%) and chrysin (97%) were purchased from Sigma-Aldrich (St. Louis, MO, USA). Dihydroresveratrol (DHRSV, >97%) and lunularin (3,4′-dihydroxydibenzyl; LUNU, >97%) were synthesized as recently reported [23]. RSV 4′-*O*-sulfate (RSV-4′S), RSV 3-*O*-glucuronide (RSV-3G), dihydroresveratrol 3-*O*-glucuronide (DHRSV-3G), and RSV 3-*O*-sulfate (RSV-3S) were obtained as described elsewhere [24]. Ultrapure Millipore water was used throughout the study.

The hard gelatin capsules contained RSV (98% purity) from *Polygonum cuspidatum* (420 mg RSV each capsule) and were manufactured by Laboratorios Admira S.L. (Alcantarilla, Murcia, Spain) following the European Union’s Good Manufacturing Practices requirements.

### 2.2. Study Design

The study protocol was conducted following the ethical recommendations of the Declaration of Helsinki and approved by the Spanish National Research Council’s Bioethics Committee (Madrid, Spain) (protocol reference 087/2020). Eligible participants were healthy subjects over 18 years of age. Exclusion criteria involved the intake of antibiotics (within a month before the study), pregnancy/lactation, history of smoking, diagnosed chronic illness, taking medication or food supplements, previous gastrointestinal surgery, habitual consumption of more than 20 g of alcohol/day, being a vegetarian, or on a weight loss regimen. The volunteers signed their informed consent before participation. This is an exploratory study with no intention of evaluating changes in clinical variables. Thus, the sample size (n = 16) was estimated according to previous pharmacokinetic studies dealing with the bioavailability and metabolism of phenolic compounds [25,26,27]. The primary outcome was the detection of RSV and (or) derived metabolites in plasma and E-EVs isolated from plasma. Secondary outcomes addressed the kinetic incorporation of RSV and (or) derived metabolites into E-EVs and whether RSV intake might stimulate E-EVs release.

**Figure 1:**
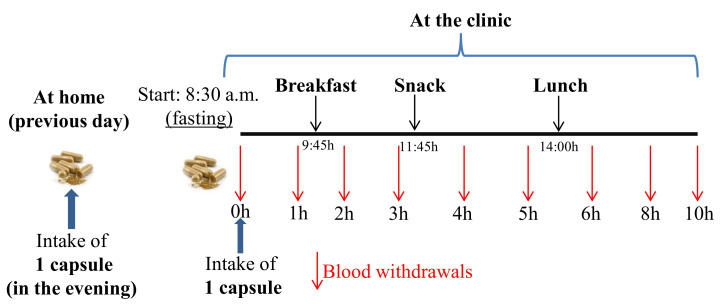
Study design.

The volunteers were instructed to follow a (poly)phenol-low diet, supervised by a Nutritionist, and based on grilled meat/fish, rice, pasta, low-fat cheese, and bread, with a reduced contribution of fruits and vegetables, legumes, nuts and seeds, juices, olive oil, coffee, wine, and tea for 1 week before the study. The participants consumed 840 mg RSV (2 capsules, 420 mg RSV each), i.e., 1 capsule (420 mg RSV) at home the evening of the day before attending the clinic to favor the presence of gut microbial-derived RSV metabolites in the bloodstream the day of the study (Figure 1), and 1 more capsule on the day of the pharmacokinetic study, before blood withdrawals. Then, they remained fasted until 9:45 h, when they ingested breakfast consisting of oat cookies and 200 mL skimmed milk. Next, they consumed a snack (cereal bar) at 11:45 and ate lunch (white rice, tuna, skimmed cheese, and sugar-free yogurt) at 14:00. Blood samples were collected at baseline (t = 0 h, before the capsule intake at the clinic) and 1, 2, 3, 4, 5, 6, 8, and 10 h after consuming the capsules (Figure 1).

### 2.3. Blood Sampling

Blood samples were collected in EDTA-coated tubes. Plasma samples were obtained by centrifugation of peripheral venous blood at 1000× *g* for 10 min at 4 °C and immediately frozen at −80 °C. Plasma samples (300 µL) were extracted with 900 μL acetonitrile–formic acid (98:2, *v*/*v*). After centrifugation at 14,000× *g* for 10 min at 4 °C, the supernatant was evaporated in a speed vacuum concentrator. Finally, the samples were resuspended in 100 μL of methanol and filtered through a 0.22 μm PVDF filter before analysis by UPLC-ESI-QTOF-MS.

### 2.4. EVs Isolation and Characterization

EVs were isolated, quantified, and size determined starting from 8 mL of plasma (time points 0, 1, 2, 3, 8, and 10 h), according to the method described by the Minimal Information for Studies of Extracellular Vesicles (MISEV) guidelines [28]. Briefly, the plasma was centrifuged at 400× *g* for 10 min at 4 °C. Next, supernatants were transferred into new tubes, and the pellets were discarded. Then, the supernatants were centrifuged at 2500× *g* for 15 min at 4 °C and 10,000× *g* for 5 min at 4 °C to remove large particles, dead cells, and cellular debris. Then, the supernatants were diluted 6 times with filtered PBS and were centrifuged at 100,000× *g* for 105 min at 4 °C. After discarding the supernatant, the pellets were carefully rinsed with PBS to remove the residual plasma contaminants and then resuspended into 550 μL of filtered PBS.

The EV concentration and size distribution curves were assessed by nanoparticle tracking analysis (NTA) using a NanoSight LM10 instrument with a 638 nm laser and NTA 3.1 software (Malvern, UK), as previously described [17]. Briefly, samples were diluted in sterile PBS to obtain the optimal detection concentration of 106–109 particles/mL, and three 60 s videos were recorded using a camera level. The particle concentrations were corrected for the input sample volume, the volume of EVs resuspension, and the dilution necessary for NTA-reading. The volume of the isolated EV fraction was estimated as previously described [29].

As reported elsewhere, EVs were processed to extract the phenolic cargo [17]. Briefly, 400 μL of PBS-resuspended EVs, isolated from the plasma time points 0, 1, 2, 3, 8, and 10 h, were mixed using ethyl acetate with 0.1% formic acid (1:4, *v*/*v*). The samples were vortexed, sonicated, and centrifuged at 14,000× *g* for 10 min at 4 °C. Then, the supernatant was reduced to dryness, and the residues were resuspended in 100 µL of methanol, filtered through a 0.22 µm PVDF filter, and injected into the UPLC-ESI-QTOF-MS.

### 2.5. Protein Determination and Western Blot Analysis

The protein concentration of EVs was determined by the Pierce™ BCA Protein Assay (Thermo Scientific, Waltham, MA, USA), using bovine serum albumin as the standard and following the manufacturer’s instructions.

The identification of EVs, including exosomes, was assessed by western blot. Protein (50 µg) was loaded for sodium dodecyl sulfate–polyacrylamide gel electrophoresis (SDS-PAGE). Membranes were blocked in 5% non-fat dry milk, following gel transfer into a nitrocellulose membrane, and incubated with the primary antibodies of the exosomal markers anti-CD63 (bs-1523R, Bioss, Woburn, MA, USA), anti-TSG101 (A303-506A, Bethyl, Montgomery, TX, USA), anti-calnexin (ab75801, Abcam, Cambridge, UK), and anti-CD9 (9PU-01MG, Immunostep, Salamanca, Spain). The secondary antibodies used were the anti-rabbit, anti-mouse, or anti-human conjugated with either Alexa FluorTM 680 or IRDye^®^ 800. Protein band images were acquired with an Odyssey^®^ infrared imaging system (LI-COR, Lincoln, NE, USA) and analyzed for image processing using the Image Studio Lite 5.2.5 software (LI-COR).

### 2.6. Analysis of Resveratrol (RSV) and Derived Metabolites

A previously validated method for analyzing RSV and its metabolites in biological samples was used [30,31]. The analyses were performed on an Agilent 1290 Infinity UPLC system coupled to a 6550 Accurate-Mass quadrupole-time-of-flight (QTOF) mass spectrometer (Agilent Technologies, Waldbronn, Germany) using an electrospray interface (Jet Stream Technology, Auburn, AL, USA).

Data were processed using the Mass Hunter Qualitative Analysis software (version B.08.00), which lists and rates possible molecular formulas consistent with the accurate mass measurement and the actual isotopic pattern. A target screening strategy was applied, searching for more than 20 possible masses of metabolites, i.e., free (unconjugated) RSV and its derived gut microbial metabolites (DHRSV and LUNU), as well as their corresponding phase-II conjugates (mainly glucuronides, sulfates, and sulfoglucuronides of RSV, DHRSV, and LUNU) that could be present in the plasma and (or) the EVs after consuming the RSV capsules [30,31]. The screening was based on mass filtering at the exact mass using a narrow mass extraction window (0.01 *m/z*), and the quantification was performed with available authentic standards. Other values are given as the area obtained from the extracted ion chromatogram (EIC) of those metabolites with no available standards.

### 2.7. Statistical Analysis

Pharmacokinetic parameters were calculated using the pharmacokinetic software PKSolver [32]. The non-compartmental pharmacokinetic parameters such as maximum concentration (C_max_) and time to reach maximum concentration (T_max_), area under the curve from 0 to t (AUC_0–24_), C_last_/C_max_ ratio, and half-life (T_1/2_) were calculated in the plasma and the EVs. The data are presented as mean ± standard deviation (SD). Significant differences in EV particle number, size, and protein content between time points were calculated using one-way ANOVA (normal distribution). Differences between plasma and EV pharmacokinetic parameters, protein concentration, and the number of particles of quantified RSV metabolites were calculated using paired ANOVA followed by Tukey’s multiple comparison test. In those quantified RSV-derived metabolites, logistic regressions and Spearman correlations were used to analyze the possible association between AUC_0–24_, T_max_, C_max_, and T_1/2_, and sex, age, and (or) BMI status in the plasma and the EVs. Differences were considered significant at *p* < 0.05.

## 3. Results

### 3.1. Volunteers’ Characteristics

The volunteers (n = 16) were all Europeans, 10 males and 6 females, with a mean (and range) age of 34.6 ± 6.9 years (26–51) and a mean body mass index (and range) of 23.8 ± 3.1 kg/m^2^ (19.1–30.7). The volunteers reported no side effects after consuming RSV capsules.

### 3.2. EVs Isolation and Characterization

**Figure 2:**
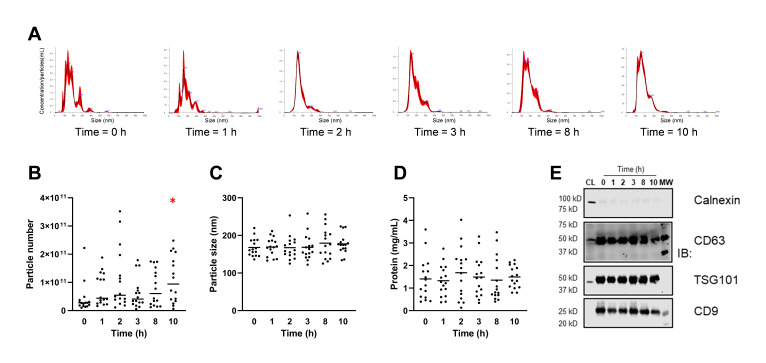
(**A**) E-EVs concentration (particles/mL) vs. size distribution (nm) by nanoparticle tracking analysis (NTA) of one volunteer at different time points; (**B**) The particle number (particles/mL of plasma) is shown on the y-axis and the different time point extraction on the x-axis; (**C**) Particle size mode (nm) at the different time points. The dots depict measurements from each volunteer, and the bars show the means; (**D**) Protein concentration; (**E**) Western blot of EV proteins (CL, cell lysate as negative control). * *p* < 0.05.

Previously rinsed with PBS to remove residual plasma, isolated EVs were subjected to NTA analysis (Figure 2). Figure 2A shows a representative NTA histogram from a volunteer at different time points after RSV intake. Despite the high variability, the particle number significantly increased at the final time point (t = 10 h) (Figure 2B). In contrast, no significant size or protein content changes were observed at the different time points analyzed (Figure 2C,D). The exosome-related protein markers were verified by western blotting, which confirmed the enrichment in CD63, CD9, and TSG101, as exosome markers, and a negligible content of calnexin, an endoplasmic reticulum contaminant (Figure 2E). As previously commented, and according to the ISEV [2], EVs can be named exosomes when these exosome markers are identified. However, we did not perform further purification steps after ultracentrifugation, and henceforth we use the term exosome-containing EVs (E-EVs) since we consider it a more rigorous term that better reflects the nature of the isolated EVs in the present study. According to the mean particle number calculated (4.9 × 10^10^ particles/mL of plasma) and the Askenase et al., (2021) estimation method, we estimated ≈60 μL E-EVs volume in 8 mL plasma.

### 3.3. Identification of RSV and Derived Metabolites

A total of 17 compounds were identified in the plasma after the analysis by UPLC-ESI-QTOF-MS. However, of 17 compounds found in the plasma, only 9 were identified in E-EVs (Table 1). Significantly, RSV metabolites with the highest molecular weights (RSV diglucuronides and RSV sulfoglucuronides) were not detected in E-EVs, although they were found in the plasma samples of all the volunteers. In addition, some sulfate conjugates from dihydroresveratrol (DHRSV) and lunularin (LUNU) only appeared in the plasma samples (Table 1). In particular, DHRSV-S (isomer-1) was only detected in the plasma samples. Regarding LUNU conjugates, while LUNU glucuronides were detected in E-EVs, only LUNU-S (isomer-1) was identified in one volunteer. Finally, free (unconjugated) RSV was not found in E-EVs, albeit traces were detected in the plasma of eight volunteers.

### 3.4. Pharmacokinetics of RSV Metabolites in Plasma and E-EVs

**Figure 3:**
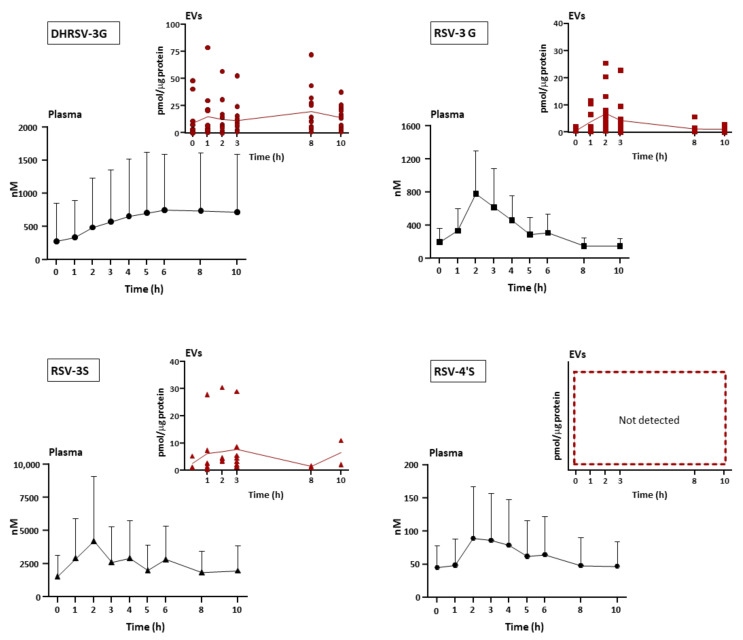
Time-course plasma and E-EVs resveratrol (RSV) metabolites concentration following RSV intake. Points at 0 h show the concentrations after 8–10 h of the first RSV dose (420 mg), and from 1 to 10 h show the profiles after the second dose (420 mg), at the beginning of the pharmacokinetic study. DHRSV, dihydroresveratrol; G, glucuronide; S, sulfate. Plasma values are shown as mean ± SD (n = 16), and the individual dot plots represent the E-EV concentration where the metabolite was quantified. Connecting lines indicate the mean values at each time point. EV concentration values are normalized by protein concentration.

Table 2 shows the pharmacokinetic parameters of those plasma and (or) E-EV metabolites quantified with authentic standards. The kinetic profiles of quantified RSV metabolites were similar in the plasma and the E-EVs (Table 2 and Figure 3). No statistically significant differences were observed when comparing the pharmacokinetics of each RSV metabolite between those in the plasma and in the E-EVs. The plasma T_max_ for RSV-3G, RSV-3S, and RSV-4′S were similar and ranged from 2.3 ± 0.7 to 2.7 ± 1.6 h, whereas the microbial metabolite DHRSV-3G, as expected, showed a higher T_max_ value (6.0 ± 2.8 h). In general, the plasma and E-EV T_max_ values were similar, except for RSV-3S, which presented a lower, but not statistically significant, T_max_ in E-EVs (1.8 ± 1.0 h) than in the plasma (2.7 ± 1.6 h) (Table 2). Consequently, the C_last_/C_max_ ratio values in the plasma and E-EVs were similar for RSV-3G and DHRSV-3G, but different for RSV-3S (Table 2). Since RSV-4′S was not detected in E-EVs, this comparison was not addressed.

Regarding C_max_ values in the plasma, the sequence from the highest to the lowest was as follows: RSV-3S > DHRSV-3G > RSV-3G > RSV-4′S. Remarkably, the sequence was slightly different in E-EVs, i.e., DHRSV-3G > RSV-3S > RSV-3G. It should be pointed out that both RSV-3S and RSV-3G reached close C_max_ values in E-EVs, even though the plasma C_max_ value of RSV-3S was approximately 7-fold higher than that of RSV-3G (Table 2 and Figure 3). In accordance with these differences, the plasma AUC_0–24_ values were RSV-3S > DHRSV-3G > RSV-3G > RSV-4′S, while in E-EVs they were DHRSV-3G > RSV-3G > RSV-3S. Therefore, these results indicate that glucuronides seem to be incorporated into E-EVs more efficiently than sulfates.

As expected, the plasma and E-EV T_1/2_ and T_max_ values of DHRSV-3G were significantly different from the rest of the metabolites (Table 3). However, regarding C_max_ values, only DHRSV-3G vs. RSV-3S showed statistical significance (*p* < 0.001) in the plasma. By contrast, in E-EVs, the differences in the C_max_ values were only observed after comparing the DHRSV-3G vs. RSV-3G pair (*p* < 0.001, Table 3). On the other hand, while the plasma RSV-3S showed higher C_max_ values than the rest of the RSV metabolites (*p* < 0.001, Table 3), no significant differences were observed in the E-EV pairs. Overall, the differences observed in the pharmacokinetic parameters for the plasma and the E-EVs with regard to DHRSV-3G point to an increase of this metabolite in E-EVs compared to RSV phase-II metabolites.

Table 4 shows the plasma/E-EVs ratios, confirming the higher DHRSV-3G concentration in E-EVs compared to RSV-3G and RSV-3S, and the similar DHRS-3G concentration in the plasma and the E-EVs. In addition, Table 4 also shows that the ratio plasma/E-EVs for RSV-3S was higher than for RSV-3G, confirming a lower RSV-3S encapsulation efficiency in E-EVs. We also compared the plasma/E-EVs ratios for all the metabolites using their EIC areas and the plasma and E-EVs volumes (i.e., Area/μL) (Supplementary Table S1). This comparison confirmed the same figures calculated in the quantified metabolites using standards, and the similar plasma/E-EVs ratios for the microbial-derived metabolites, especially in the case of glucuronides. In addition, DHRSV-S (isomer-2) was detected in all the plasma samples but only appeared in the E-EVs from half of the volunteers. In contrast, DHRSV-3G appeared in all the volunteers’ plasmas and E-EVs.

Regarding LUNU, its conjugates were detected only in 8 of 16 volunteers. LUNU-G (isomer-1) and LUNU-S (isomer-1) were detected in most plasma samples from the lunularin-producing volunteers. Interestingly, LUNU S (isomer-1) was detected in E-EVs only in one volunteer, whereas LUNU G (isomer-1) was detected in five out of the six volunteers in plasma. Therefore, the results obtained for DHRSV and LUNU conjugates also support the conclusion that sulfates seemed to be less efficiently encapsulated into E-EVs than glucuronides, despite sulfates being similar or more abundant in the plasma than glucuronides. Finally, the kinetic profiles of DHRSV and LUNU conjugates in the plasma and the E-EVs showed a sustained detection of these metabolites in the different assay times analyzed (Supplementary Figure S1).

We also explored the possible influence of volunteers’ sex, age, and (or) BMI on the kinetic parameters of those RSV metabolites quantified in the plasma and the E-EVs. We observed a negative correlation between AUC_0–24_ and BMI for DHRSV-3G in E-EVs (r = −0.62, *p* = 0.02). Furthermore, also for DHRSV, C_max_ and BMI were inversely correlated in both the plasma (r = −0.51, *p* = 0.046) and E-EVs (r = −0.60, *p* = 0.025). No other statistical associations were found. Nevertheless, further studies with a larger sample size should confirm or discard these possible associations. In addition, the threshold RSV dose to detect metabolites in E-EVs should be explored in further dose–response studies in which the sensitivity of the analytical method will be crucial.

## 4. Discussion

Despite the generally-accepted health benefits of (poly)phenols, it is still unclear whether the effects are exerted by the ingested phenolics and (or) their microbial metabolites and (or) their corresponding phase-II conjugates. In addition, it is not fully known whether the effects always require a direct interaction between the (poly)phenol or metabolite and the target cell and (or) can be mediated by indirect signaling cascades [9,33]. In this context, EVs, including exosomes, have been suggested as possible mediators of signals elicited by (poly)phenols, or as nanocarriers that could contribute to their transport to systemic targets, including the brain [15,17,34]. Thus, in the puzzle of (poly)phenols and health, we provide here the first evidence on the transport of resveratrol (RSV) metabolites by E-EVs in humans.

In a previous study, we did not detect any (poly)phenol metabolite in the plasma exosomes from fasting subjects following a standard diet (not supplemented with (poly)phenols) [7]. Thus, we designed the present pharmacokinetic trial to answer two challenging questions, i.e., (i) the (poly)phenol dose needed to allow the detection of derived metabolites (if any) in human E-EVs, and (ii) the kinetics of metabolites’ encapsulation.

In this trial, the first RSV dose consumed by the volunteers was the evening before the pharmacokinetic trial, to enhance the production of the gut microbial-derived metabolites and their possible encapsulation into E-EVs, since a single RSV intake, and the follow-up in a pharmacokinetic study, could not be enough to detect microbial-derived metabolites. Despite the high inter-individual variability observed, the kinetic profiles of RSV metabolites were quite similar in the plasma and the E-EVs, with no significant differences in the kinetic parameters of metabolites quantified with standards. Furthermore, plasma RSV diglucuronides and sulfoglucuronides were not detected in E-EVs, suggesting that molecular size matters in E-EVs encapsulation. Finally, the plasma concentration of sulfate derivatives was higher than that of glucuronides, with the opposite being the case in E-EVs, which deserves further research to investigate the possible associated mechanisms resulting in the differential encapsulation of glucuronides and sulfates into E-EVs. In addition, while RSV metabolites were preferentially transported in the plasma, showing higher plasma/E-EVs ratios, the concentration of the gut microbial-derived metabolites was similar in E-EVs and plasma, especially in the case of glucuronides. Since EVs can target systemic organs and deliver their cargo efficiently [17,34,35], our results might provide additional clues to clarify the gap between the bioavailability of (poly)phenols and their health effects [9]. In this regard, these results could have additional relevance in the context of neuroprotection, considering the ability of EVs to cross the blood–brain barrier, and thus favoring the passage of certain (poly)phenols to the brain [36,37,38].

We also explored the possible occupancy ratio of RSV metabolites and E-EVs. In contrast to that observed for certain molecules (i.e., miRNAs), where there is a large excess of exosomes compared to the total number of possible miRNAs [39], our stoichiometric analysis suggested that most individual E-EVs could transport a large amount of RSV metabolites molecules (results not shown). Indeed, our data are consistent with a high-occupancy/high-RSV metabolites concentration model (considering the number of particles of ≈6.02214076 × 10^23^/mol of a given substance), in which a significant fraction of E-EVs carries a high concentration of RSV metabolites. Nevertheless, this issue requires further research since other models cannot be discarded, including the low-occupancy/high-RSV metabolites concentration model, in which E-EVs in the population carry many particles of a given RSV metabolite.

All cells can release EVs, but identifying the exact origin of all EV fractions is challenging [3], and this was not addressed in the present study. It is known that RSV reaches the colon, where it is metabolized [24]. However, unconjugated RSV, DHRSV, and LUNU were not detected in E-EVs. Notably, in the case of LUNU, its presence in E-EVs will depend on the subjects’ gut microbiota. While all individuals can produce DHRSV, not all (approximately 75%) can produce LUNU. In this regard, two metabotypes associated with RSV metabolism by the human gut microbiota, i.e., LUNU-producers and LUNU non-producers, have been recently reported [40].

Since only phase-II RSV metabolites, including the microbial ones, were detected in E-EVs, we can speculate that most E-EVs could originate from enterocytes and hepatocytes, where most phase-II conjugations occur. However, we cannot discard a possible different encapsulation efficiency between free RSV and their conjugates in E-EVs, although free RSV has been reported to be encapsulated in milk exosomes by passive diffusion [17].

The present study has not determined the possible presence of EVs secreted by the gut microbiota, and this requires further investigation. For example, on the one hand, since only phase-II metabolites were detected in E-EVs, but not unconjugated RSV and its derived gut microbial metabolites produced in the colon, we speculate that most circulating E-EVs containing RSV metabolites were not of microbial origin. However, on the other hand, we cannot discard the possibility that conjugated metabolites accessible to the gut microbiota through the enterohepatic circulation could then be encapsulated by EVs secreted by the gut microbiota.

Since this is the first human study on (poly)phenol transport in EVs, we can only compare our results with those reported in a previous rat study [18]. However, in addition to the different species, i.e., rat vs. human, the (poly)phenols assayed were different (grape proanthocyanidins), as well as the derived metabolites (flavanols and low-weight phenolics), and only two time points were analyzed [18]. That rat study reported a significant amount of (poly)phenolic compounds and derived metabolites in EVs isolated from plasma using precipitation kits. In addition, the metabolic profile observed in the plasma and the EVs was identical. In contrast, when ultracentrifugation was employed as the isolation method, no (poly)phenols were found in EVs [18]. A likely explanation for these results is that ultracentrifugation separates plasma EVs with lower yields but higher purity than precipitation reagents [41]. In addition, EVs isolated with precipitation kits can precipitate other plasma proteins, including those that bind phenolics, which could justify the identical metabolic profile in the plasma and the EVs. For these reasons, we used ultracentrifugation instead of precipitation kits in the present study. We acknowledge that separation exclusion chromatography (SEC) is more appropriate to purify exosomes, but even so, the presence of other EVs cannot be excluded [17]. In the present study, the number of samples and plasma volume prevented the use of SEC as a routine purification protocol. Nevertheless, since the metabolic profile observed in the plasma and the E-EVs was different, and considering the western blot results, we can conclude the isolated EV fractions contained exosomes (E-EVs) but not residual plasma.

Finally, intriguing evidence suggests that (poly)phenols can act on signaling pathways that interfere with the biogenesis of EVs. For instance, grape polyphenols can reduce platelet- and endothelial-derived microparticles production [42]. Moreover, the increased levels of endothelial microparticles observed in coronary artery disease patients were reduced by cocoa flavanol consumption [43]. In contrast, curcumin promoted exosomal secretion in cell lines [11,12,13]. In the present study, despite the high variability, we observed a significant change in the number of E-EVs after RSV supplementation only at the final time point analyzed (t = 10 h), which suggested that chronic RSV consumption could exert a more sustained effect on E-EVs release. Nevertheless, this issue deserves further research.

In summary, this is the first human study on (poly)phenol transport in EVs, using RSV to illustrate it. Whether other (poly)phenols and (or) their gut microbial-derived metabolites are also transported in E-EVs requires further research. In addition, the differential encapsulation of glucuronides vs. sulfates and the similar metabolite concentration in E-EVs vs. plasma for the gut microbial-derived metabolites are relevant issues. However, this needs further confirmation for other (poly)phenols. Finally, we have provided additional clues on the bioavailability and tissue disposition of RSV and derived metabolites using E-EVs as in vivo nanocarriers, which pave the way for investigating the possible role of E-EVs in (poly)phenol health effects.

## Figures and Tables

**Table 1:** Resveratrol (RSV) and its derived metabolites in plasma (P) and (or) exosome-containing extracellular vesicles (E-EVs).

Compound	RT (min)	Mass Accuracy (*m/z^−^*)	Molecular Formula	Error (ppm)	Score	Occurrence
RSV-diG (isomer-1)	4.42	579.1355	C_26_H_28_O_15_	−1.95	97.31	P
RSV-diG (isomer-2)	4.89	579.1355	C_26_H_28_O_15_	−1.85	97.46	P
RSV-SG (isomer-1)	5.21	483.0603	C_20_H_20_O_12_S	−1.15	98.68	P
RSV-SG (isomer-2)	5.76	483.0603	C_20_H_20_O_12_S	0.10	99.15	P
RSV-4′G	6.27	403.1035	C_20_H_20_O_9_	0.01	99.57	P, E-EVs
RSV-4′S *	7.57	307.0282	C_14_H_12_O_6_S	−1.96	98.01	P
DHRSV-4′G	7.66	405.1191	C_20_H_22_O_9_	0.45	99.24	P, E-EVs
RSV-3G *	7.71	403.1035	C_20_H_20_O_9_	−0.03	97.50	P, E-EVs
DHRSV-S (isomer-1)	8.12	309.0438	C_14_H_14_O_6_S	−2.63	92.35	P
DHRSV-3G *	8.40	405.1191	C_20_H_22_O_9_	−2.41	97.45	P, E-EVs
RSV-3S *	8.72	307.0282	C_14_H_12_O_6_S	−2.85	94.23	P, E-EVs
DHRSV-S (isomer-2)	8.81	309.0438	C_14_H_14_O_6_S	1.51	97.51	P, E-EVs
RSV *	10.94	227.0714	C_14_H_12_O_3_	0.07	90.87	P
LUNU-G (isomer-1)	11.54	389.1242	C_20_H_22_O_8_	−3.45	94.95	P, E-EVs
LUNU-G (isomer-2)	11.62	389.1242	C_20_H_22_O_8_	0.73	99.36	P, E-EVs
LUNU-S (isomer-1)	11.64	293.0489	C_14_H_14_O_5_S	−1.55	91.21	P, E-EVs
LUNU-S (isomer-2)	12.18	293.0489	C_14_H_14_O_5_S	−3.53	90.93	P

* Identification using authentic standards. The rest of the compounds were tentatively identified according to their exact molecular mass, high score (>90), and low error (<5 ppm). RT, retention time. DHRSV, dihydroresveratrol; LUNU, lunularin; G, glucuronide; S, sulfate; diG, diglucuronide; SG, sulfoglucuronide.

**Table 2:** Pharmacokinetic parameters of resveratrol (RSV) metabolites * in the plasma samples and E-EVs.

Metabolites	T_1/2_ (h)		T_max_ (h)		C_max_		C_last_/C_max_		AUC_0–24_	
	Plasma	E-EVs	Plasma	E-EVs	Plasma (nM)	E-EVs (pmol/µg protein)	Plasma	E-EVs	Plasma (nM·h)	E-EVs (pmol/µg protein·h)
DHRSV-3G	13.0 ± 11.9	11.7 ± 12.2	6.0 ± 2.8	5.9 ± 3.9	902 ± 895	28.1 ± 22.0	0.67 ± 0.31	0.55 ± 0.32	6196 ± 7702	144 ± 132
RSV-3S	4.8 ± 2.4	^#^	2.7 ± 1.6	1.8 ± 1.0	6481 ± 5387	10.5 ± 11.5	0.36 ± 0.20	0.66 ± 0.37	30,208 ± 24391	19.2 ± 19.4
RSV-4′S	6.9 ± 2.2	–	2.6 ± 1.2	–	271 ± 688	–	0.47 ± 0.11	–	1881 ± 5011	–
RSV-3G	4.1 ± 2.2	3.1 ± 1.8	2.3 ± 0.7	2.6 ± 2.2	843 ± 530	9.0 ± 8.5	0.23 ± 0.13	0.29 ± 0.34	3510 ± 2050	26.2 ± 23.6

* Quantified with authentic standards. DHRSV, dihydroresveratrol; G, glucuronide; S, sulfate; –, not detected in E-EVs; T_max_, time of maximum concentration; T_1/2_, time required for the concentration to reach half of its original value; C_max_, maximum concentration; C_last_/C_max_ ratio between last observed (quantifiable) concentration (C_last_) and C_max_; AUC_0–24_, area under the curve from the time of dosing to the final measurable concentration (24 h). ^#^ Not determined due to the concentration of terminal phase showing an ascending tendency in most samples.

**Table 3:** Comparison of plasma and E-EV pharmacokinetic parameters among the different quantified RSV metabolites.

	T_1/2_ (h)		T_max_ (h)		C_max_		C_last_/C_max_		AUC_0–24_	
	Mean Diff.	*p*	Mean Diff.	*p*	Mean Diff.	*p*	Mean Diff.	*p*	Mean Diff.	*p*
**Metabolite pairs in plasma**										
RSV-3S vs. RSV-4′S	−2.11	ns	0.17	ns	6209	**<0.001**	28327	**<0.001**	−0.12	ns
RSV-3S vs. RSV-3G	0.71	ns	0.42	ns	5638	**<0.001**	26698	**<0.001**	0.13	ns
RSV-3S vs. DHRSV-3G	−8.21	**0.003**	−3.26	**<0.001**	5579	**<0.001**	24012	**<0.001**	−0.32	**<0.001**
RSV-4′S vs. RSV-3G	2.83	ns	0.25	ns	−571.1	ns	−1629	ns	0.24	**0.005**
RSV-4′S vs. DHRSV-3G	−6.09	**0.03**	−3.44	**<0.001**	−630.8	ns	−4315	ns	−0.19	**0.03**
RSV-3G vs. DHRSV-3G	−8.92	**<0.001**	−3.69	**<0.001**	−59.69	ns	−2686	ns	−0.44	**<0.001**
**Metabolite pairs in E-EVs**										
RSV-3S vs. RSV-3G	–	–	−0.86	ns	4.29	ns	0.33	ns	−5.178	ns
RSV-3S vs. DHRSV-3G	–	–	−4.08	**0.005**	−14.82	ns	0.07	ns	−123.0	**0.004**
RSV-3G vs. DHRSV-3G	–	**0.03 ^a^**	−3.214	**0.01**	−19.11	**0.008**	−0.2647	ns	−117.8	**0.002**

Significant differences are shown in bold (Tukey’s post hoc tests for multiple comparisons); –, not determined; ^a^ Value from the Mann–Whitney U test; ns, not significant.

**Table 4:** RSV metabolite concentration (μM) in both plasma and E-EVs ^a^.

	Time Points												
Metabolites	0 h		1 h		2 h		3 h		8 h		10 h		
	Plasma	E-EVs	Plasma	E-EVs	Plasma	E-EVs	Plasma	E-EVs	Plasma	E-EVs	Plasma	E-EVs	Mean Plasma/E-EVs (-Fold)
**RSV-3G**	0.2 ± 0.2	0.04 ± 0.03	0.3 ± 0.3	0.1 ± 0.2	0.8 ± 0.5	0.2 ± 0.3	0.6 ± 0.4	0.1 ± 0.1	0.1 ± 0.1	0.04 ± 0.04	0.15 ± 0.09	0.03 ± 0.03	
Plasma/E-EVs (-fold)	**5.2**		**3**		**4**		**6**		**2.5**		**5**		**4.3 ± 1.3**
**RSV-3S**	1.6 ± 1.6	0.1 ± 0.03	2.9 ± 2.9	0.2 ± 0.3	4.2 ± 4.8	0.4 ± 0.6	2.6 ± 2.7	0.2 ± 0.1	1.9 ± 1.6	0.1 ± 0.04	1.9 ± 1.8	0.3 ± 0.3	
Plasma/E-EVs (-fold)	**16**		**14.5**		**10.5**		**13**		**19**		**6.3**		**13.2 ± 4.4**
**DHRSV-3G**	0.3 ± 0.6	0.3 ± 0.6	0.3 ± 0.5	0.4 ± 0.4	0.5 ± 0.7	0.4 ± 0.3	0.6 ± 0.8	0.4 ± 0.4	0.7 ± 0.8	0.5 ± 0.3	0.7 ± 0.8	0.6 ± 0.4	
Plasma/E-EVs (-fold)	**1**		**0.7**		**1.2**		**1.5**		**1.4**		**1.2**		**1.1 ± 0.3**

^a^ Values are shown as mean ± SD. Plasma volume = 8 mL; E-EVs volume ≈60 μL. The time point 0 h shows the concentrations after 8–10 h of the first RSV dose (420 mg), and from 1 to 10 h the concentrations after the second dose (420 mg) at the beginning of the pharmacokinetic study. DHRSV, dihydroresveratrol; G, glucuronide; RSV, resveratrol; S, sulfate.

## Data Availability

Not applicable.

## Supplementary Materials

The following are available online at https://www.mdpi.com/article/10.3390/nu14173632/s1, Figure S1: Kinetic profiles of dihydroresveratrol (DHRSV) and lunularin (LUNU) conjugates in plasma and E-EVs. Points at 0 h show the concentrations after 8–10 h of the first RSV dose (420 mg), and from 1 to 10 h show the profiles after the second dose (420 mg), at the beginning of the pharmacokinetic study. Areas of the integrated extracted ion chromatograms (EICs) are used as relative quantification. Plasma values are shown as mean ± SD (n = 16), and the individual dot plots represent the E-EV concentration where the metabolite was quantified. Connecting lines indicate the mean values at each time point. E-EV concentration values are normalized by protein concentration. G, glucuronide; S, sulfate. Table S1: RSV metabolite concentration (EIC Area/μL) in plasma and E-EVs.
