# Supplementary material for: Exosome-Containing Extracellular Vesicles Contribute to the Transport of Resveratrol Metabolites in the Bloodstream: A Human Pharmacokinetic Study
Source: Nutrients. 2022 Sep 2;14(17):3632. doi: 10.3390/nu14173632 (PMC9459822; doi:10.3390/nu14173632)
Supplement: Supplementary file 1 [file nutrients-14-03632-s001.zip › Figure S1.pdf]

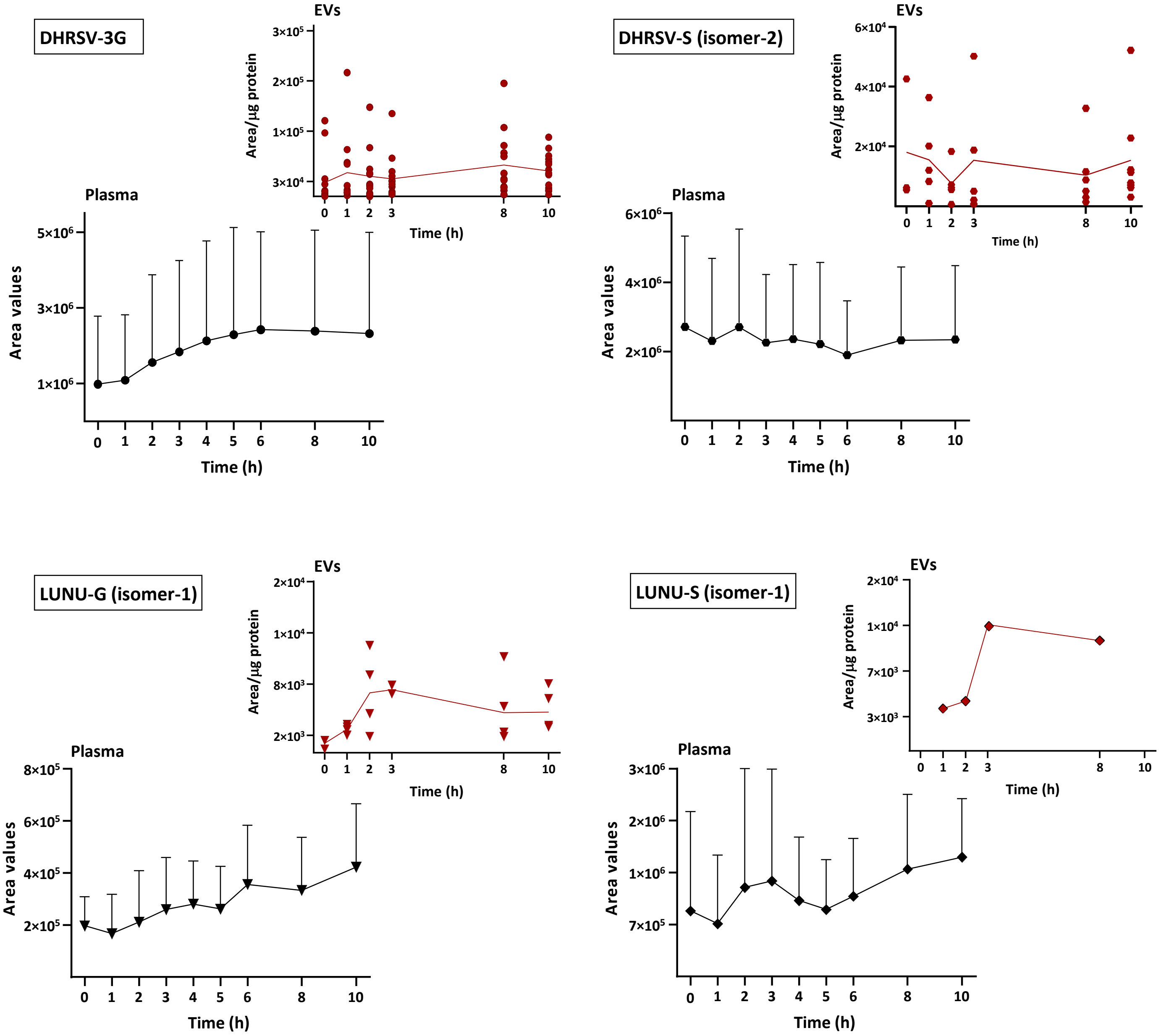

Figure S1. Kinetic profiles of dihydroresveratrol (DHRSV) and lunularin (LUNU) conjugates in plasma and E-EVs. Points at 0 h show the concentrations after 8 10 h of the first RSV dose (420 mg), and from 1 to 10 h show the profiles after the second dose (420 mg), at the beginning of the pharmacokinetic study. Areas of the integrated extracted ion chromatograms (EICs) are used as relative quantification. Plasma values are shown as mean ± SD (n = 16), and the individual dot plots represent the E-EV concentration where the metabolite was quantified. Connecting lines indicate the mean values at each time point. E-EV concentration values are normalized by protein concentration. G, glucuronide; S, sulfate.
