# Supplementary material for: Exosome-Containing Extracellular Vesicles Contribute to the Transport of Resveratrol Metabolites in the Bloodstream: A Human Pharmacokinetic Study
Source: Nutrients. 2022 Sep 2;14(17):3632. doi: 10.3390/nu14173632 (PMC9459822; doi:10.3390/nu14173632)
Supplement: Supplementary file 1 [file nutrients-14-03632-s001.zip › Table S1.pdf]

Table S1. RSV metabolite concentration (EIC Area / μL) in plasma and E-EVs [a].

Metabolites	Time points												
	0 h		1 h		2 h		3 h		8 h		10 h		
	Plasma	E-EVs	Plasma	E-EVs	Plasma	E-EVs	Plasma	E-EVs	Plasma	E-EVs	Plasma	E-EVs	Mean Plasma/E-EVs (-fold)
**RSV-3G**	5599±4418	1296±1166	8501±7151	4022±6159	19973±13819	6743±10005	17536±11777	4646±5433	4756±3572	1483±1267	4308±2688	742±529	
Plasma/E-EVs (-fold)	***4.3***		***2.1***		***3***		***3.8***		***3.2***		***5.8***		***3.7±1.7***
**RSV-4′G**	7673±6326	3139±1790	10095±7653	5253±5813	19989±12884	13672±11464	26351±30284	9540±7313	6671±4289	3207±3441	6347±3846	2082±1759	
Plasma/E-EVs (-fold)	***2.4***		***1.9***		***1.5***		***2.8***		***2.1***		***3***		***2.3±0.6***
**RSV-3S**	48938±37451	3118±845	77189±72839	5689±9144	120760±111533	12479±18615	75264±59311	6163±4023	48420±39385	2204±1306	53154±45138	8653±8561	
Plasma/E-EVs (-fold)	***15.6***		***13.6***		***9.7***		***12.2***		***21.9***		***6.1***		***13.1±5.4***
**DHRSV-3G**	3257±6000	3649±7569	3611 ±5773	5398±5967	5173±7723	4937±4510	6116±8054	5291±5619	7952±8893	6280±4114	7720±8922	7948±5035	
Plasma/E-EVs (-fold)	***0.9***		***0.7***		***1.04***		***1.2***		***1.3***		***0.97***		***1.0±0.2***
**DHRSV-4′G**	1900±4865	1440±1394	1764±3996	766±663	2519±4636	458±306	2042±3290	605±541	1848±4246	1300±2449	1592±2838	676±243	
Plasma/E-EVs (-fold)	***1.3***		***2.3***		***5.5***		***3.4***		***1.4***		***2.3***		***2.7±1.5***
**DHRSV-S (isomer 2)**	9022±8769	2731±3043	7674±7962	1442±833	9009±9442	976±570	7508±6581	1274±1081	7739±7089	1403±716	7807±7118	1857±947	
Plasma/E-EVs (-fold)	***3.3***		***5.3***		***9.2***		***5.9***		***5.5***		***4.2***		***5.5±2.2***
**LUNU-G (isomer-1)**	657±370	245±69	557±502	463±183	702±661	737±253	866±666	660±147	1111±678	613±420	1407±814	750±653	
Plasma/E-EVs (-fold)	***2.7***		***1.2***		***0.95***		***1.3***		***1.8***		***1.9***		***1.6±0.6***
**LUNU-G (isomer-2)**	554±698	452[b]	540±634	843±757	654±780	1378±1359	781±884	962±625	871±719	1019±156	1155±898	1196±49	
Plasma/E-EVs (-fold)	***1.2***		***0.6***		***0.5***		***0.8***		***0.85***		***0.96***		***0.8±0.2***
**LUNU-S (isomer-1)**	657±370	–	557±502	308[b]	702±661	337[b]	866±666	916[b]	1111±678	800[b]	1407±814	–	
Plasma/E-EVs (-fold)			***1.8***		***2.1***		***0.94***		***1.4***				***1.5±0.5***

[a]Values are shown as mean ± SD. EIC Area / μL allows the relative comparison of plasma/E-EVs ratios for all the metabolites. Plasma volume = 8 mL; E-EVs volume ≈ 60 μL. [b]No SD or range is shown because it was detected only in one volunteer.–, not detected. The time point 0 h shows the concentrations after 8–10 h of the first RSV dose (420 mg), and from 1 to 10 h the concentrations after the second dose (420 mg) at the beginning of the pharmacokinetic study. DHRSV, dihydroresveratrol; G, glucuronide; LUNU, lunularin; RSV, resveratrol; S, sulfate. RSV-4′S was not detected in E-EVs.
